# The cut-off values of handgrip strength and lean mass index for sarcopenia among patients on peritoneal dialysis

**DOI:** 10.1186/s12986-020-00506-3

**Published:** 2020-10-08

**Authors:** Xiao Xu, Zhikai Yang, Tiantian Ma, Ziqian Li, Yuan Chen, Yingdong Zheng, Jie Dong

**Affiliations:** 1 Renal Division, Department of Medicine, Peking University First Hospital; Institute of Nephrology, Peking University; Key Laboratory of Renal Disease, Ministry of Health; Key Laboratory of Renal Disease, Ministry of Education; Research Units of Diagnosis and Treatment of Immune-Mediated Kidney Diseases, Chinese Academy of Medical Sciences, Beijing, China; 2 Clinical Nutrition Department, Peking University First Hospital, Beijing, China; 3 Department of Epidemiology and Biostatistics, School of Public Health, Peking University, Beijing, China

**Keywords:** Hand grip strength, Lean mass index, Sarcopenia, Mortality, Peritoneal dialysis, Chronic kidney disease

## Abstract

**Background:**

Sarcopenia is common and contributes to a high risk of mortality among general population. There is no consensus regarding the cut-off values for sarcopenia in terms of mortality among chronic kidney disease patients. This study aimed to explore and validate cut-off points of handgrip strength (HGS) and lean mass index (LMI) for estimating the risk of mortality in peritoneal dialysis (PD) patients.

**Methods:**

This single-center prospective cohort study enrolled 1089 incident PD patients between October 2002 and July 2019. All patients were followed until death, transfer to hemodialysis, receiving renal transplantation or the end date of study (December 2019). All participants were randomly sampled to development cohort (70% participants) and validation cohort (30% participants), matched by gender and diabetes. Lean body mass was calculated by using the equation published by our center. Cubic spline regression analysis was used to examine the relationship between HGS or LMI values and mortality, and explore the cut-off points after adjusting for age, diabetes, cardiovascular disease and serum albumin in the development cohort. The derived cut-off values were verified by the agreement rate for predicting mortality and then compared with cut-off values from various clinical guidelines in the validation cohort.

**Results:**

All 1089 patients were followed up with the median of 36.0 (18.0, 71.0) months. In the development cohort, cut-off points for predicting the higher mortality were derived as 24.5 kg and 14 kg of HGS for males and females, 16.7 kg/m^2^ and 13.8 kg/m^2^ of LMI for males and females respectively. In the validation cohort, these cut-off values significantly predicted worse outcomes, with HR 1.96 (1.35, 2.84) of HGS and HR 1.76 (1.26, 2.47) of LMI for all-cause mortality after multivariate adjustment. The newly derived cut-off points of HGS have numerically higher prognostic values in all-cause mortality compared with those from current clinical guidelines, and agreement rates of HGS were 65.2 versus 62.5–64.6 respectively.

**Conclusions:**

The derived cut-off values of HGS and LMI have sufficient and better prognostic value in predicting all-cause mortality in PD patients compared with the cut-off values in the existing guidelines. These cut-off values are only validated in a single population, thus limiting the generalizability.

## Introduction

Sarcopenia, which is defined as age-associated loss of skeletal muscle mass and function [1–4] is associated with progressive worsening of nutritional and clinical conditions, as well as a high risk for morbidity and mortality [1, 5–7]. Recently, there has been an increasing amount of published evidence on the high risk of sarcopenia in patients with chronic kidney disease (CKD) [8–10], and the subsequent increase in mobility disability [11], frailty [12], cardiovascular events [13], and mortality [13, 14]. In dialysis patients, the uremic environment leads to an increase in protein catabolism that results in diminished muscle mass and function and accelerates the development of sarcopenia [15–17]. Therefore, early diagnosis and treatment of sarcopenia is important in patients with all stages of CKD, especially patients on dialysis.

Several guidelines have published the cut-off value of hand grip strength (HGS) and lean mass index (LMI) to determine the sarcopenia [2–4, 18]. These values were derived from epidemiological measures, i.e. the lower 20th percentile [3] or 2–2.5 standard deviation (SD) [2, 18] below the mean value in the general population, and rarely been verified from the prognostic perspective. Several studies performed in dialysis patients have used the cut-off values recommended by these guidelines to report the prevalence of sarcopenia, 11–41% [16, 19–22], and indicated close associations between sarcopenia and hospitalization [19], cardiovascular disease (CVD) [23, 24] and mortality [19, 25]. These data implicated the importance of screening sarcopenia but did not evaluate the appropriateness of these cut-off values.

Before we determine the definition of sarcopenia for dialysis patients, cut-off values of HGS and LMI must be derived from this population, especially it is noted that the distribution of HGS and LMI in CKD patients is different from that in normal individuals [25–29]. In the field of nephrology, the therapeutic targets of anemia [30] and mineral and bone disorder [31] have ever been derived from epidemiological data on the relationship of cut-off values of hemoglobin [32, 33], serum phosphorus [34, 35], or intact parathyroid hormone [34, 36] and clinical outcomes. A similar approach could be used to define the HGS and LMI cut-off values for screening the sarcopenia among dialyzed individuals who are at high risk for death.

Therefore, in the present study, we aimed to define and validate cut-off values of HGS and LMI for predicting all-cause mortality through a single-center longitudinal peritoneal dialysis (PD) cohort. The predictive value of newly-derived cut-off points of HGS would be also compared with existing HGS cut-off values recommended by guidelines from general population [2–4, 18].

## Materials and methods

### Subjects and follow-up

This is a prospective cohort with data retrospectively analyzed, carried out at the PD center of Peking University First Hospital. All incident PD patients between October 1, 2002, and July 31, 2019 were screened. Patients were excluded if they refused to complete the baseline test, denied the diagnosis of end-stage renal disease or could not be regularly followed. All participants were followed until death, transfer to hemodialysis (HD), renal transplantation, loss to follow-up or the end of study (Dec. 31, 2019). All subjects began the PD program within 1 month after catheter implantation and were given lactate-buffered glucose dialysate (Baxter Healthcare, Guangzhou, China). Among them, 96.7% patients were treated with continuous ambulatory peritoneal dialysis. This study was approved by the Medical Ethics Committee of Peking University (Number: 2018 research 100). Written informed consent was obtained from each patient.

### Demographic and clinical data

Age, gender, body mass index, CVD, diabetes mellitus (DM) were collected within the week preceding PD catheter implantation. CVD was recorded if 1 of the following conditions was present: angina; class III–IV congestive heart failure (as defined by the New York Heart Association); transient ischemic attack; history of myocardial infarction or cerebrovascular accident; peripheral arterial disease [37]. Baseline values were recorded as mean measurements of blood pressure, biochemistry data, dialysis adequacy, and nutrition parameters during the first 3 months. The nutrition patameters including biochemical data, i.e. albumin, serum lipids and serum potassium, anthropometry data such as height, weight, HGS, and estimated LMI. More details are as follows.

### Biochemical, and dialysis adequacy variables

Biochemistry data including hemoglobin, serum albumin, lipids spectrum, uric acid, urea, creatinine, calcium, phosphate, and intact parathyroid hormone were examined using an automatic Hitachi chemistry analyzer (Hitachi Chemical, Tokyo, Japan). Serum high-sensitive C-reactive protein (hs-CRP) was measured by immune rate nephelometric analysis. Dialysis adequacy and residual renal function were measured by collecting 24-h urine and dialysate. Dialysis adequacy was defined as total urea clearance and total creatinine clearance. Residual renal function was estimated using the average renal clearance of urea and creatinine.

### HGS and LMI

Standing height was measured using a fixed stadiometer and weight using a calibrated digital scale. HGS was measured using an adjustable handheld dynamometer. The dynamometer was held freely, without support. Then participants were told to put maximal force on the dynamometer. Three consecutive measurements of HGS (in kg) by both hands were averaged [38]. HGS evaluated in the dominant arm was used to develop the prediction equation. Lean body mass (LBM) was calculated using the following formulas for CKD stage 3–5 patients derived by us: LBM (kg) = (1 if male; 0 if female) × 4.72 + height (cm) × 0.28 + weight (kg) × 0.27 + HGS (N) × 0.02—dialysis duration (months) × 0.04—26.84 [39]. As compared with the gold standard, dual-energy X-ray absorptiometry, LBM estimated from this equation has shown small bias and better accuracy than LBM from creatinine kinetics and anthropometry methods. LMI (kg/m^2^) was calculated as LBM divided by the square of height. Baseline values of HGS and LMI were calculated as the mean HGS and LMI during the first 3 months.

### Definition of outcome event

The outcome was all-cause death. In all analyses, all patients were followed to death, transfer to HD, renal transplantation, loss to follow-up, or the end of the study (Dec 31, 2019).

### Statistical analysis

Parametric data are presented as mean ± standard deviation. Nonparametric data are presented as median values with an inter-quartile range (IQR). Categorical variables are expressed as percentages or ratios. All participants were randomly sampled to the development cohort and the validation cohort matched by gender and DM. 70% participants were selected into development cohort and 30% into validation cohort. Student's t test, nonparametric tests or the Χ^2^ test was used to compare the differences of variables between cohorts as appropriate.

To develop the cut-off points for defining the sarcopenia, Cubic spline regression analysis was used in male and female respectively to examine the relationship between baseline HGS or LMI values and mortality, and then explore the cut-off point of HGS (HGS-spline) and cut-off point of LMI (LMI-spline) for a higher mortality after adjusting for recognized confounders including age, DM, CVD, and serum albumin. To investigate the functional form, we constructed natural piecewise-cubic spline functions with the specified sequence of interior knots placed at the 20, 40, 60, 80 points of the distributions of HGS and LMI, respectively. In the spline figures, we brought 5–95% of the HGS and LMI distribution in analysis to avoid incredible extrapolation from extreme data.

To validate the newly-derived cut-off values, HGS-spline, LMI-spline were evaluated by Cox proportional regression model respectively, adjusting for age, CVD, DM, and serum albumin. Survival functions of participants in above COX modals were used to predict the outcome of patients (survival or death). The percentage of absolute agreement was reported and estimated by kappa statistics comparing the predictive outcome and the real outcome.

The percentage of absolute agreement of the HGS cut-off values in existing international guidelines were reported in the same way and compared with the newly-derived cut-off values. The guidelines include the European Working Group on Sarcopenia in Older People (EWGSOP) 2010 [2], EWGSOP update in 2019 (EWGSOP 2019) [18], the Asian Working Group for Sarcopenia (AWGS) [3] and the Foundation for the National Institutes of Health Biomarkers Consortium Sarcopenia Project (FNIH) [4] guidelines.

All probabilities were two-tailed and the level of significance was set at 0.05. Hazard ratios (HRs) and 95% confidence intervals (CIs) were calculated using competing risk survival analysis. Statistical analysis was performed using SPSS software package version 24.0 (SPSS, Chicago, IL, USA) and SAS version 9.4 (SAS Institute, Cary, NC, USA).

## Results

### Baseline characteristics and follow-up

**Fig. 1:**
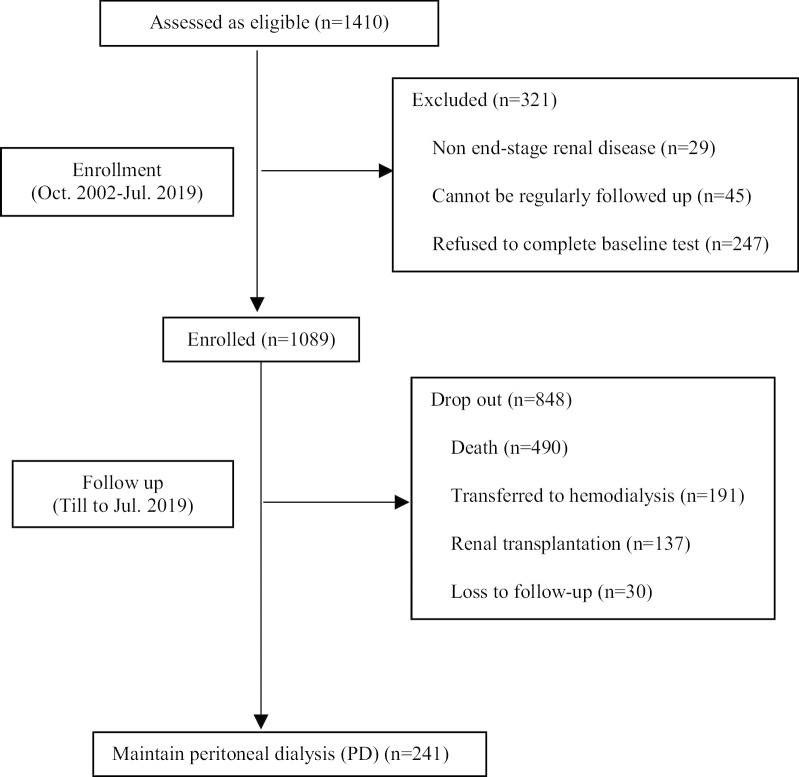
Flow chart of the study

We screened 1410 incident PD patients between October 2002 and July 2019. In total 1089 incident PD patients were enrolled, with mean age of 56.4 ± 11.1 years, 50.8% being men. The prevalence of DM and CVD was 38.9% and 39.5% respectively. The mean follow-up time was 36 [17, 17] months (Fig. 1). The other 321 patients were excluded based on the exclusion criteria.

The baseline HGS and LMI was 22.0 ± 10.9 kg and 15.6 ± 1.9 kg/m^2^ respectively. The whole cohort was randomly divided into development cohort (n = 762) and validation cohort (n = 327). The baseline characteristics of development cohort and validation cohort was shown in Table 1. The baseline HGS was 22.0 ± 10.9 kg in development cohort and 21.8 ± 10.7 kg in validation cohort. The LMI was 15.6 ± 1.9 kg/m^2^ in development cohort and 15.6 ± 2.0 kg/m^2^ in validation cohort. According to the results, there were no significant differences in demographic data, laboratory measurements, and nutritional indices between these two cohorts (*P* > 0.05).

**Table 1:** Baseline clinical characteristics of PD patients (n = 1089)

Variates	Total (n = 1089)	Development cohort (n = 762)	Validation cohort (n = 327)	*P*
Age (years)^a^	56.4 ± 11.1	56.5 ± 15.2	56.1 ± 15.0	0.640
Male, n (%)	554 (50.8)	382 (50.1)	172 (52.4)	0.509
BMI (kg/m^2^)	23.2 ± 3.8	23.3 ± 3.8	23.0 ± 4.0	0.146
DM, n (%)	424 (38.9)	297 (39.0)	127 (38.7)	0.946
CVD, n (%)	431 (39.5)	293 (38.5)	138 (42.1)	0.280
Height (cm)	164.3 ± 8.5	164.3 ± 8.5	164.4 ± 8.7	0.867
Weight (kg)	62.1 ± 12.5	62.1 ± 12.0	62.1 ± 13.5	0.947
Handgrip strength (kg)	22.0 ± 10.9	22.0 ± 10.9	21.8 ± 10.7	0.664
Lean body mass (kg)	42.6 ± 8.5	42.6 ± 9.4	42.7 ± 8.9	0.917
Lean mass index (kg/m^2^)	15.6 ± 1.9	15.6 ± 1.9	15.6 ± 2.0	0.900
Laboratory and nutrition data				
Serum albumin (g/L)	35.6 ± 4.6	35.4 ± 4.7	35.8 ± 4.4	0.188
Hemoglobin (g/L)	101.8 ± 15.8	101.7 ± 16.0	102.2 ± 15.4	0.677
Hs-CRP (mg/L)	2.2 (0.8, 6.1)	2.2 (0.9, 6.1)	2.2 (0.7, 6.1)	0.994
Urea nitrogen (mmol/L)	23.0 ± 6.3	23.0 ± 6.5	22.9 ± 6.0	0.959
Serum creatinine (umol/L)	712.6 ± 247.5	710.7 ± 247.3	717.0 ± 248.3	0.700
Serum calcium (mmol/L)	2.20 ± 0.2	2.2 ± 0.2	2.2 ± 0.2	0.131
Serum phosphorus (mmol/L)	1.6 ± 0.4	1.6 ± 0.4	1.6 ± 0.4	0.949
Serum sodium (mmol/L)	139.2 ± 2.6	139.1 ± 2.6	139.2 ± 2.6	0.531
Serum potassium (mmol/L)	4.4 ± 0.6	4.4 ± 0.6	4.4 ± 0.6	0.687
HDL-cholesterol (mmol/L)	1.1 ± 0.3	1.1 ± 0.3	1.1 ± 0.4	0.299
LDL-cholesterol (mmol/L)	2.6 ± 0.8	2.6 ± 0.8	2.6 ± 0.8	0.874
Total cholesterol (mmol/L)	4.8 ± 1.2	4.9 ± 1.3	4.8 ± 1.1	0.244
Triglycerides (mmol/L)	1.5 (1.1, 2.1)	1.5 (1.1, 2.0)	1.5 (1.2, 2.2)	0.239
iPTH (pg/mL)	170.0 (82.3, 329.5)	178.9 (84.0, 336.7)	158.6 (76.8, 313.3)	0.246
Total CCr (L/w/1.73 m^2^)	69.3 ± 28.8	68.8 ± 28.4	70.4 ± 29.6	0.432
Total Kt/V	1.9 ± 0.6	1.9 ± 0.6	1.9 ± 0.6	0.646
RRF (ml/min)	3.2 (1.7, 5.4)	3.2 (1.8, 5.1)	3.5 (1.5, 5.8)	0.345

^a^Values are expressed as a mean ± standard deviation, percentage or median with upper and lower quartile or percentage

*PD* peritoneal dialysis, *BMI* body mass index, *DM* diabetes mellitus, *CVD* cardiovascular disease, *Hs-CRP* high-sensitive C-reactive protein, *HDL* high density lipoprotein, *LDL* low density lipoprotein, *iPTH* intact parathyroid hormone, *Total CCr* total creatinine clearance, *Total Kt/V* total urea clearance, *RRF* residual renal function

At the end of the study, 241 patients were still maintained on PD, 490 died, 191 transferred to HD, 137 received renal transplantation, and 30 lost to follow-up (Table 2). The development cohort did not show any differences in mortality, transferring to HD or receiving renal transplantation, as compared to validation cohort (*P* > 0.05). With regards to causes for transferring to HD, patients in the validation cohort had a lower chance for transferring to HD due to socioeconomic issue (0.2 vs 0.8 event rate /100 person-years, *P* = 0.025). The risk for all causes of death was not significantly different between cohorts (*P* > 0.05).

**Table 2:** Outcomes and causes among PD Patients (n = 1089)

Outcomes, no. of events (event rate /100 person-years)	Total (n = 1089)	Development cohort (n = 762)	Validation cohort (n = 327)	*P*
Follow-up, months	36.0 (18.0, 71.0)	36.5 (18.0, 71.0)	35.0 (16.0, 72.0)	0.710
Death	490 (11.1)	346 (11.1)	144 (11.0)	0.888
Cardiovascular events	197 (4.5)	139 (4.5)	58 (4.4)	0.407
Infection	121 (2.7)	95 (3.1)	26 (2.0)	0.219
Tumor	43 (1.0)	27 (0.9)	16 (1.2)	0.150
Severe malnutrition	23 (0.5)	13 (0.4)	10 (0.8)	0.060
Others	105 (2.4)	72 (2.3)	33 (2.5)	0.643
Transfer to hemodialysis	191 (4.3)	138 (4.4)	53 (4.1)	0.549
PD-related infection	109 (2.5)	74 (2.4)	35 (2.7)	0.508
Fluid overload	15 (0.3)	12 (0.4)	3 (0.2)	0.216
Inadequate solute clearance	13 (0.3)	8 (0.3)	5 (0.4)	0.661
Leak	11 (0.2)	8 (0.3)	3 (0.2)	0.852
Socioeconomic issue	27 (0.6)	24 (0.8)	3 (0.2)	0.025
Others	15 (0.3)	11 (0.4)	4 (0.3)	0.051
Renal transplantation	137 (3.1)	89 (2.9)	48 (3.7)	0.172
Lost to follow-up	30 (0.7)	20 (0.6)	10 (0.8)	0.646

### The development of cut-off values of HGS and LBM

**Fig. 2:**
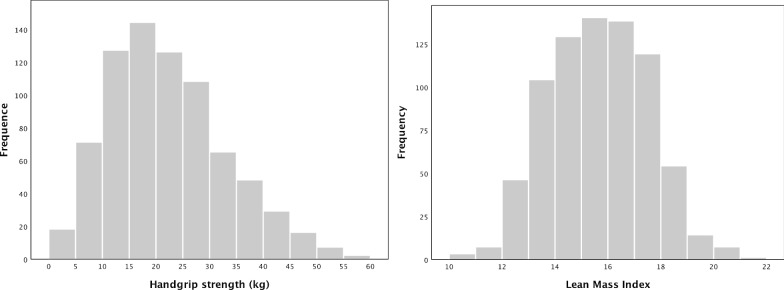
Distributions of handgrip strength and lean mass index in the development cohort

The distributions of HGS and LMI in the development cohort were showed in Fig. 2. By spline regression analysis, approximate L or S-shaped associations were observed for HGS or LMI and all-cause mortality in the development cohort. After adjusting for age, DM, CVD, and serum albumin, patients with HGS lower than 24.5 kg in male or 14.0 kg in female had a significantly higher mortality risk than patients with higher HGS; similarly, patients with LMI lower than 16.7 kg/m^2^ in male or 13.8 kg/m^2^ in female also were associated with a higher death risk than those with higher LMI (Additional file 1: Supplement Figure). These figures showed linear relationships between HGS or LMI and mortality. When analyzed as continuous variables in the COX regression analysis, each 1 kg of increase in HGS was associated with 3% of reduction in all-cause mortality, and each 1 kg/m^2^ of increase in LMI correlated to 9% of reduction in all-cause mortality after multivariate adjustment (Table 3).

**Table 3:** The prognostic value of HGS and LMI for all-cause mortality in the development cohort (n = 762)

Variables	Model-HGS			Model-LMI		
	Coefficient	HR (95% CI)	*P*	Coefficient	HR (95% CI)	*P*
Age	0.039	1.04 (1.03, 1.05)	< 0.001	0.044	1.05 (1.04, 1.06)	< 0.001
DM	0.395	1.49 (1.20, 1.84)	< 0.001	0.437	1.55 (1.24, 1.93)	< 0.001
CVD	0.454	1.57 (1.26, 1.97)	< 0.001	0.501	1.65 (1.32, 2.06)	< 0.001
Serum albumin	− 0.002	1.00 (1.00, 1.00)	0.165	− 0.002	1.00 (1.00, 1.00)	0.101
HGS, per 1 kg increase	− 0.027	0.97 (0.96, 0.99)	< 0.001			
LMI, per 1 kg/m^2^ increase				− 0.093	0.91 (0.86, 0.97)	0.003

*DM* diabetes mellitus, *CVD* cardiovascular disease, *HGS* hand grip strength, *LMI* lean mass index, *M* male, *F* female

### The validation of cut-off value of HGS and LMI

According to the newly derived cut-off values of HGS and LMI, the prevalence of sarcopenia is 26.3% in the validation group. To validate the prognostic value, we divided patients into high and low group according to newly derived cut-off value (HGS-spline and LMI-spline) from the development cohort. As shown in Table 4, patients with lower HGS or LMI had a significantly higher risk for mortality, with adjusted HR [1.96 (1.35, 2.84), *P* < 0.001] and [1.76 (1.26, 2.47), *P* = 0.002] respectively. The total agreement rate of the prognostic mortality by HGS-spline and LMI-spline was 65.2% and 62.5%, respectively, compared with the real outcome.

**Table 4:** The prognostic value of cut-off points of HGS and LMI for all-cause mortality in the validation cohort (n = 327)

Groups	Model 1		Model 2	
	HR (95% CI)	*P*	HR (95% CI)	*P*
HGS (kg) groups				
≥ 24.5 (M)/14.0 (F) (n = 183)	Reference		Reference	
< 24.5 (M)/14.0 (F) (n = 144)	3.37 (2.37, 4.80)	< 0.001	1.96 (1.35, 2.84)	< 0.001
LMI (kg/m^2^) groups				
≥ 16.7 (M)/13.8 (F) (n = 195)	Reference		Reference	
< 16.7 (M)/13.8 (F) (n = 132)	1.91 (1.37, 2.65)	< 0.001	1.76 (1.26, 2.47)	0.001
HGS and LMI groups				
HGS ≥ 24.5 (M)/14.0 (F) and LMI ≥ 16.7 (M)/13.8 (F) (n = 143)	Reference		Reference	
LMI < 16.7 (M)/13.8 (F) (n = 51)	1.24 (0.66, 2.34)	0.501	1.49 (0.79, 2.82)	0.217
HGS < 24.5 (M)/14.0 (F) (n = 47)	3.00 (1.87, 4.80)	< 0.001	1.72 (1.05, 2.80)	0.030
HGS < 24.5 (M)/14.0 (F) and LMI < 16.7 (M)/13.8 (F) (n = 86)	3.70 (2.45, 5.59)	< 0.001	2.49 (1.61, 3.85)	< 0.001

Model 1: non-adjusted;

Model 2: adjusted for age, DM, CVD, and serum albumin

*HGS* hand grip strength, *LMI* lean mass index, *M* male, *F* female, *DM*, diabetes mellitus, *CVD* cardiovascular disease

We further divided patients into four groups based on both cut-off values of HGS and LMI. As compared to patients with both higher HGS and LMI, those with lower HGS alone, or combined with lower LMI had significantly higher mortality with HR 1.72 (1.05, 2.80) and HR 2.49 (1.61, 3.85) respectively. Patients with lower LMI alone did not increase the risk for death compared with those with both higher HGS and LMI. These data verified the prognostic value of cut-off points of both HGS and LMI, but HGS cut-off value having more influence on predicting the mortality.

### Comparisons in the prognostic value of difference cut-off values

According to HGS-spline and the existing HGS cut-off values in guidelines, the percentage of patients with lower HGS was 41.9% versus 51.1–75.2%. Compared with the cut-off values in existing guidelines, the predictive value of HGS-spline for mortality was numerically highest in the validation cohort (Table 5). The absolute agreement rate (%) for the HGS-spline was 65.2 (Kappa = 0.27), which was numerically higher than those for cut-off values of HGS recommended by several existing guidelines, 64.6 for HGS-EWGSOP 2019, 63.7 for HGS-GWAS, 62.8 for HGS-FNIN, 62.5 for HGS-EWGSOP 2010 (Kappa, 0.25–0.21).

**Table 5:** Agreement rate of mortality according to different cut-off values

Validation group (n = 327)	Cut-off value (male/female)	Percentage of patients with lower HGS/LMI (%)	Total agreement rate (%)	Kappa
HGS-spline	24.5/14.0 (kg)	41.9	65.2 (60.1, 70.4)	0.265 (0.161, 0.369)
HGS-EWGSOP 2019	27.0/16.0 (kg)	51.1	64.6 (59.4, 69.8)	0.248 (0.146,0.350)
HGS-GWAS	26.0/18.0 (kg)	55.0	63.7 (58.5, 68.9)	0.233 (0.129, 0.337)
HGS-FNIH	26.0/16.0 (kg)	57.5	62.8 (57.5, 68.0)	0.214 (0.110, 0.318)
HGS-EWGSOP 2010	30.0/20.0 (kg)	75.2	62.5 (57.2, 67.7)	0.210 (0.104, 0.316)
LMI-spline	16.7/13.8 (kg/m^2^)	40.4	62.5 (57.2, 67.7)	0.213 (0.107, 0.319)
HGS and LMI-spline	HGS: 24.5/14.0 (kg) LMI: 16.7/13.8 (kg/m^2^)	26.3	64.0 (58.8, 69.2)	0.237 (0.133, 0.341)

*HGS-spline* cut-off values of HGS from the spline regression, *HGS-EWGSOP 2019* cut-off values of HGS from the guideline EWGSOP 2019, *HGS-GWAS* cut-off values of HGS from the guideline GWAS, *HGS-FNIH* cut-off values of HGS from the guideline FNIN, *HGS-EWGSOP 2010* cut-off values of HGS from the guideline EWGSOP 2010, *LMI-spline* cut-off values of LMI from the spline regression, *HGS and LMI-spline* cut-off value based on the both HGS and LMI from the spline regression

The absolute agreement rate for the LMI-spline was 62.5 (Kappa = 0.21). Further combined the cut-off value of LMI with that of HGS, the agreement rate was still lower (64, Kappa = 0.24) than the cut-off value of HGS alone.

## Discussion

Through this single-center prospective cohort, we first determined the cut-off values of HGS (24.5 kg in males and 14.0 kg in females) and LMI (16.7 kg/m^2^ in males and 13.8 kg/m^2^ in females) for the diagnosis of sarcopenia in PD patients using cubic spline regression analysis. We further validated the predictive role of the cut-off points of HGS and LMI from a prognostic perspective. Our data also showed that our HGS cut-off values had comparable, even better prognostic value for all-cause mortality than those recommended by existing guidelines for the diagnosis of sarcopenia [2–4, 18].

The distribution of muscle mass and strength in CKD patients is different from that in normal individuals [25–29]. Compared with the general population, the HGS of CKD patients was 5–15 kg lower [25–27] and LMI was more than 2 kg/m^2^ lower [40–43]. According to the cut-off values of HGS and LMI for the diagnosis of sarcopenia in the general population recommended by existing guidelines, the prevalence of sarcopenia in dialyzed patients is 11–68% [21, 22, 44, 45]. This wide range partially represented the lack of commonly-recognized definition for the diagnosis of low muscle mass and low muscle strength [21, 22]. On the other hand, sarcopenia defined in the healthy population may not appropriate in the dialysis population. Several studies have shown that sarcopenia by most definitions was not significantly associated with mortality after adjustment for covariates [16, 25]. For example, a previous study by Kittiskulnam et al. [25] found that sarcopenia defined by EWGSOP 2010 did not associated with a higher risk of death in HD patients. Several studies also found low muscle mass, regardless of the indexing method, lack of association with mortality in dialysis patients [16, 19, 25].

Based on the evidence on strong associations of HGS or LMI values with all-cause mortality in the development and validation cohort, our newly derived cut-off values of HGS and LMI would be helpful in screening for sarcopenia, and thereby, evaluating the risk of poor outcome in each PD subject in our clinical practice. Further, potential interventions for the improvement of muscle mass and strength, i.e., resistance exercise [46–48] and oral nutritional supplementation [49, 50], should be initiated in a timely manner in those with sarcopenia.

Compared with the cut-off values reported by the current guidelines, HGS-spline seemed to have comparable even greater power for predicting mortality, with a total agreement rate of 65.2. The current HGS cut-off value best matched the values reported by the updated EWGSOP 2019 guidelines [18] (HGS < 27 kg in males and < 16 kg in females, which were 2.5 SD below the gender-specific peak mean in the European population). Although the race of subjects in the AWGS report was consistent with that of our patients [3], the predictive effect of the HGS cut-off value by AWGS was weaker than that of EWGSOP 2019 guidelines and our HGS cut-off value, when the agreement rate associated with mortality was considered. All existing guidelines seemed to overestimate the prevalence of sarcopenia in PD patients given that cut-off values of HGS are higher than our, even the best matched one (EWGSOP 2019) having a 1.5–2 kg deviation compared with HGS-spline.

Most previous studies have confirmed that HGS is a stronger predictor of mortality than LBM in dialysis patients [16, 19, 25]. For example, Isoyama et al. [16] reported low HGS was more strongly associated with mortality than low muscle mass. Also, there was a research that found muscle mass lack of association with mortality [25]. Consistent with these studies, we also found the predictive effect of HGS to be better than that of LMI with regard to mortality in dialysis patients. In the multivariate analyses, patients with lower LMI alone (< 16.7 kg/m^2^ for male, 13.8 kg/m^2^ for female) did not increase the risk for death compared with those with both higher HGS (≥ 24.5 kg for male, 14.0 kg for female) and higher LMI (≥ 16.7 kg/m^2^ for male, 13.8 kg/m^2^ for female). The agreement tests also showed that the cut-off value of HGS had a higher agreement rate than that of LMI, and HGS combine with LMI in predicting the death risk. These findings indicated that LMI played an obviously weaker role in the prediction of mortality than HGS in dialysis patients. Further, the HGS test is more convenient and simpler, and entails a lower cost than the LBM or LMI test. The updated EWGSOP 2019 guidelines also considered low muscle strength as the primary parameter for the diagnosis of sarcopenia [18], and stressed on the greater value of HGS, rather than LBM, for assessing sarcopenia. For these reasons, we recommend that HGS be applied as a routine screening test in everyday clinical practice.

The present study has several strengths. To the best of our knowledge, it is the first study to explore the cut-off value of HGS and LMI for predicting all-cause mortality in PD patients. Additionally, we have validated the cut-off values in a validation cohort (independent of the development cohort), and this further verified its effectiveness in PD patients. Furthermore, the study was performed in a large PD cohort with a relatively long follow-up period and sufficient endpoints. Finally, repeat measurements, including HGS and LMI, in the first three months of PD provided a reliable evaluation at the baseline.

We have to acknowledge certain limitations of our study, too. The main limitation is that we did not use the gold standard for evaluating LBM. As a result, there might have been some difference between the estimated value and the actual value of LMI. However, LBM estimation with our previously developed equations has been verified as a precise method with a very small bias compared with dual-energy X-ray absorptiometry [39], a commonly recognized standard examination. Another limitation was the observational design of the study, we therefore cannot exclude the possibility that unrecognized factors confounded the observed associations between exposure, i.e., HGS or LMI, and mortality. Nonetheless, we did adjust for the most important confounders, such as age, gender, DM, CVD, and serum albumin, while exploring the key associations. The third limitation is the single-center study design, which limits the generalizability of our data. In addition, we did not evaluate the changes in HGS or LMI and their association with mortality. Only absolute values rather than the changes in these indices could be applied for the diagnosis of sarcopenia. Finally, further interventions to improve muscle mass and strength should be performed to verify whether any gained effects would be achieved once the patients’ HGS or LMI are increased to above the cut-off values.

## Conclusions

In conclusion, based on development and validation process, we concluded that PD patients with weak HGS (< 24.5 kg in male and < 14.0 kg in female), and decreased LMI (< 16.7 kg/m^2^ in male and < 13.8 kg/m^2^ in female) have sufficient high risk on all-cause mortality through a prospective PD cohort with relatively large sample size and long follow up. The definition of sarcopenia according to these values should be verified further in a larger PD population, taking into their precision and reliability in the diagnosis and treatment of sarcopenia. In the current state, our newly-derived cut-of values of HGS and LMI, especially HGS as a simpler measure, are helpful in screening the sarcopenia and evaluating the risk for adverse outcomes in PD patients.

## Supplementary information

**Additional file 1: Supplement Figure**. Association between HGS or LMI and adjusted hazard of mortality.

## Data Availability

All data generated or analysed during this study are included in this published article.

## Abbreviations

HGS: Handgrip strength
LMI: Lean mass index
PD: Peritoneal dialysis
CKD: Chronic kidney disease
SD: Standard deviation
CVD: Cardiovascular disease
HD: Hemodialysis
DM: Diabetes mellitus
hs-CRP: High-sensitive C-reactive protein
LBM: Lean body mass
IQR: Inter-quartile range
EWGSOP: European Working Group on Sarcopenia in Older People
AWGS: Asian Working Group for Sarcopenia
FNIH: Foundation for the National Institutes of Health Biomarkers Consortium Sarcopenia Project
HRs: Hazard ratios
CIs: Confidence intervals
