# Supplementary material for: The cut-off values of handgrip strength and lean mass index for sarcopenia among patients on peritoneal dialysis
Source: Nutr Metab (Lond). 2020 Oct 8;17:84. doi: 10.1186/s12986-020-00506-3 (PMC7542899; doi:10.1186/s12986-020-00506-3)
Supplement: Supplementary file 1 — Additional file 1: Supplement Figure. Association between HGS or LMI and adjusted hazard of mortality. [file 12986_2020_506_MOESM1_ESM.docx]

Supplement figure. Association between HGS or LMI and adjusted hazard of mortality

A

**Ln(HR) where the ref values for HGS is 24.5 kg**

**HGS (kg)**

B

**Ln(HR) where the ref values for HGS is 14.0 kg**

**HGS (kg)**

**HGS (N)**

C

**Ln(HR) where the ref values for LMI is 16.7kg/m^2^**

**LMI (kg/m^2^)**

D

**Ln(HR) where the ref values for LMI is 13.8kg/m^2^**

**LMI (kg/m^2^)**

Models were performed using spline regression analysis with knots at the 20th, 40th, 60th and 80th percentiles. Solid line represents estimated Ln(HR), dotted line represents the 95% CI. Models are adjusted for age, DM, CVD and serum albumin. (A) HGS in male; (B) HGS in female; (C) LMI in male; (D) LMI in female.

Abbreviation: HGS, hand grip strength; LMI, lean mass index.
